# First trimester complete blood cell indices in early and late onset preeclampsia

**DOI:** 10.4274/tjod.galenos.2019.93708

**Published:** 2019-07-03

**Authors:** Gökçen Örgül, Duygu Aydın Haklı, Gonca Özten, Erdem Fadiloğlu, Atakan Tanacan, Mehmet Sinan Beksaç

**Affiliations:** 1 Hacettepe University Faculty of Medicine, Department of Obstetrics and Gynecology, Division of Perinatology, Ankara, Turkey; 2 Hacettepe University Faculty of Medicine, Department of Biostatistics, Ankara, Turkey

**Keywords:** Early onset preeclampsia, late onset preeclampsia, complete blood count, white blood cell count, neutrophil count

## Abstract

**Objective:**

This study aimed to compare the first trimester complete blood count (CBC) indices of pregnancies complicated by early-onset preeclampsia (EOPE) or late-onset preeclampsia (LOPE).

**Material and Methods:**

A retrospective case-control study was conducted with 186 patients. Patients were classified into three subgroups: EOPE, LOPE, and control groups. First trimester CBC results were obtained for each patient. Hemoglobin, hematocrit, red blood cell distribution width, mean corpuscular volume, white blood cell (WBC) count, neutrophil, eosinophil, basophil, lymphocyte, monocyte, mean platelet volume, platelet distribution width, plateletcrit, and platelet count were compared. The neutrophil lymphocyte ratio was calculated by dividing the absolute lymphocyte count by the absolute neutrophil count. The platelet lymphocyte ratio was calculated by dividing the absolute lymphocyte count by the absolute platelet count.

**Results:**

The total number of cases was 21, 42, and 123, in the EOPE, LOPE, and control groups, respectively. There were statistically significant differences in the total WBC and neutrophil counts between the three groups (both p<0.05). WBC and neutrophil counts were found to be highest in the EOPE group, and the LOPE group had higher levels compared with controls. The optimal cut-off values to predict EOPE for WBC and neutrophil counts were 9.55×10^3^/ μL (sensitivity 71.4% and specificity 70.7%) and 6.45×10^3^/μL (sensitivity 66.7% and specificity 74.8%), respectively.

**Conclusion:**

Increased first trimester WBC and neutrophil counts may be predictive for EOPE.

**PRECIS:** The optimal cut-off values to predict early onset preeclampsia for white blood cells and neutrophil counts were 9.55×10^3^/μL (sensitivity 71.4% and specificity 70.7%) and 6.45×10^3^/μL (sensitivity 66.7% and specificity 74.8%).

## Introduction

Preeclampsia is an important public health problem that is defined as new onset hypertension and proteinuria after the 20^th^ gestational week. Hypertensive disorders of pregnancy occur in almost 10% of all pregnancies, and the prevalence of preeclampsia is approximately 3-5% worldwide^(1,2)^. Recently, preeclampsia was classified into two subgroups according to the onset time of clinical findings and symptoms. Early-onset preeclampsia (EOPE) refers to disease that occurs before the 34^th^ gestational week, whereas after 34 weeks of pregnancy, the disease is commonly referred to as late-onset preeclampsia (LOPE)^(3,4)^. The prevalence of LOPE is reported to be almost seven-fold higher than that of EOPE (2.72% vs. 0.38%)^(4)^.

Early- and late-onset preeclampsia are the consequences of different underlying pathophysiologic conditions. Placentation defects, which are attributable to defective syncytiotrophoblast invasion and abnormal remodeling of the spiral arteries, are associated with early-onset disease. On the other hand, LOPE usually occurs in the presence of maternal endothelial dysfunction, which is associated with more favorable neonatal outcomes compared with EOPE. Briefly, EOPE seems to be a placental disorder, whereas LOPE is typically linked to maternal factors^(3,5)^.

The complete blood count (CBC) is a cheap and easily accessible test that shows the cellular elements of the blood. This simple test provides valuable information about the immune system. The distribution of the different blood cell components was reported to be relevant in assessing certain obstetric complications including preeclampsia, preterm delivery, and preterm premature rupture of membranes^(6,7)^. Parameters such as leukocytosis, the neutrophil lymphocyte ratio (NLR), the platelet lymphocyte ratio (PLR), mean platelet volume (MPV), and red blood cell distribution width (RDW) have been shown to be associated with inflammation, and various studies have reported changes in these values according to disease processes^(8,9,10)^.

Many studies in the literature have focused on comparing first trimester CBC indices between severe and mild preeclampsia^(11,12)^. However, we decided to conduct our study based on the onset of preeclampsia, which is a new and popular topic in perinatal medicine. Therefore, the objective of this study was to show the first trimester CBC parameter differences in pregnancies complicated by EOPE and LOPE.

## Materials and Methods

This was a retrospective case-control study of 186 pregnant women who gave birth at Hacettepe University Hospital between January 2012 and December 2017. The study was approved by the Hacettepe University Non-Interventional Clinical Researches Ethics Board (approval no: GO18/358). The study was conducted in accordance with the World Medical Association Declaration of Helsinki regarding the ethical conduct of the research.

Preeclampsia diagnosis was made in accordance with the 2013 report of the American College of Obstetricians and Gynecologists, as follows: (i) new onset of hypertension (systolic blood pressure ≥140 mm Hg or diastolic blood pressure ≥90 mm Hg) after 20 weeks of gestation in a previously normotensive patient and (ii) proteinuria on urinalysis (≥300 mg/24 hour or dipstick ≥1+) or (iii) end-organ symptoms/findings (thrombocytopenia, altered serum creatinine level, increased liver transaminases, pulmonary edema, or cerebral or visual symptoms)^(13) ^. Preeclampsia diagnosed before the 34^th^ gestational week was defined as EOPE, while it was defined as LOPE after the 34^th^ gestational week^(3)^.

The study subjects were divided into the EOPE, LOPE, and control groups according to the criteria explained above. The control group was randomly selected from the computerized hospital database. This group was composed of healthy pregnancies without any maternal or fetal complications (e.g. gestational diabetes, preterm delivery, congenital malformations). Pregnant women with chronic diseases, cigarette smoking, alcohol consumption or any drug use were excluded from the study. Gestational age was estimated by the last menstrual period and confirmed using the first trimester crown-rump length measurement.

First trimester CBC testing is a routine part of first trimester follow-up in our institution. Venous blood samples were transferred into K2-EDTA tubes, and a UniCel DXH800 fully automated cell counter (Beckman Coulter Inc., US) was used for the CBC. The results of the red cell parameters, including hemoglobin (Hb), hematocrit (Htc), RDW, mean corpuscular volume (MCV), the white blood cell (WBC) count and subtypes (neutrophil, eosinophil, basophil, lymphocyte, and monocyte), and the thrombocyte indices (MPV, platelet distribution width (PDW), plateletcrit, and platelet count) were collected for each patient from the computerized hospital database. The NLR was calculated by dividing the absolute lymphocyte count by the absolute neutrophil count. PLR was calculated by dividing the absolute lymphocyte count by the absolute platelet count. If there were two or more CBC results for the same patient, we used the earliest set. Maternal age, obstetric history, gestational week at birth, birth weight, and APGAR scores were also collected from the patient files.

### Statistical Analysis

Statistical analyses were performed using the Statistical Package for the Social Sciences version 22 software package (SPSS, Chicago). The Kolmogorov-Smirnov test was used to analyze the distribution of data. Variables are given as median (minimum-maximum) or mean ± standard deviation. The three groups were compared using the Kruskal-Wallis test or one-way analysis of variance. Statistical significance was defined as a p value less than 0.05. In the EOPE group, receiver operating characteristic curves (ROC) were generated, and the area under the curve (AUC) was calculated for significant values. The cut-off, sensitivity, and specificity were calculated using the ROC curves and Youden’s index.

## Results

There were 21 cases in the EOPE group, 42 cases in the LOPE group, and 123 cases in the control group. The demographic characteristics of the patients, delivery times, and neonatal findings are shown in Table 1. There were no statistically significant differences between the three groups in terms of maternal age, body mass index (BMI), and obstetric history. However, gestational age at birth, birth weight, and APGAR scores were lower in the EOPE group.

The number of babies with a 1^st^ minute APGAR score below seven was 13 (61.9%) in the EOPE group, 3 (7.1%) in the LOPE group, and 3 (2.4%) in the control group. The number of babies with a 5^th^ minute APGAR score below seven was 7 (33.3%) in the EOPE group, 1 (2.4%) in the LOPE group, and 0 in the control group.

In the LOPE group, 45.2% of the cases (n=19) were born between 34 and 37 weeks; the remaining 23 (54.8%) cases were born after the 37^th^ gestational week. All neonates in the EOPE group weighed <2500 g, whereas all babies in the control group weighed >2500 g. In the LOPE group, the distribution of cases according to birth weight was 33.3% (n=14) at <2500 g, 61.9% (n=26) at 2500-4000 g, and 4.8% (n=2) at >4000 g.

Table 2 shows the findings of the first trimester CBC parameter results in each group. There were no statistically significant differences between the three groups in terms of the Hb, Htc, MCV, RDW, eosinophils, basophils, monocytes, lymphocytes, and plateletcrit. However, there was a statistically significant difference in the WBC and neutrophil counts between the three groups. The WBC and neutrophil counts were highest in the EOPE group, and the LOPE group had higher levels compared with the control group. Although the MPV and PDW values differed between the three groups, a pairwise analysis did not demonstrate any significant differences between the three groups, as shown in Table 3.

**Figure 1:**
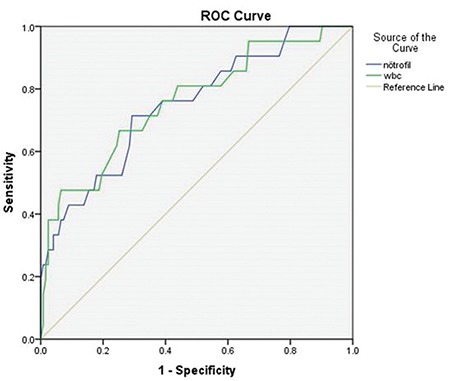
The performance of white blood cells and neutrophils to identify early onset preeclampsia ROC: Receiver operating characteristic, WBC: White blood cell

The performance of the two variables (WBCs and neutrophils) was used to identify the EOPE and control groups using the ROC analysis as shown in Figure 1. For the WBC and neutrophil counts, the AUC was 0.758 (95% CI: 0.633-0.866) and 0.749 (95% CI: 0.639-0.877), respectively, and the p values were <0.05 for both parameters. The optimum cut-off values for the WBCs and neutrophils were 9.55×10^3^/µL (sensitivity 71.4% and specificity 70.7%) and 6.45×10^3^/µL (sensitivity 66.7% and specificity 74.8%), respectively.

## Discussion

Diagnosis of preeclampsia commonly refers to the classic findings of new-onset hypertension and proteinuria in pregnant women after 20 weeks of gestation. On the other hand, EOPE and LOPE are now considered as two different disorders with unincorporated pathophysiologies. Impaired placental development in early pregnancy is mainly associated with EOPE, whereas maternal vascular instability due to endothelial injury is more typically associated with LOPE. Increased inflammation resulting from these different dysfunctions is the mutual effect, and such inflammatory changes/processes are thought to be responsible for the maternal and fetal complications^(3,14)^.

Pregnancy is associated with certain hematologic changes such as decreased Hb, increased MCV, leukocytosis, neutrophilia, and slight thrombocytopenia^(15)^. Although the most common laboratory finding in preeclampsia is proteinuria, other changes (thrombocytopenia, neutrophilia, and Htc increase) may also be detected in a routine CBC^(16)^. Accordingly, investigators have conducted several studies to investigate the potential identification or prediction of preeclampsia and disease severity using first trimester hemogram results^(11,12,17)^. To the best of our knowledge, no English language article in the current literature has compared first trimester CBC values between patients with EOPE and LOPE.

Anemia is a common health problem, especially in low income countries; however, a recent systematic meta-analysis reported that anemia during the first and second trimester was not associated with preeclampsia^(18)^. In our study, we were also unable to find any differences in anemia parameters (Hb, Htc, MCV) between the three groups. RDW is another red cell parameter in routine CBC testing that indicates the variation in erythrocyte cell sizes. Increased RDW levels, a condition referred to as anisocytosis, are shown to be linked to inflammation in the general population. Furthermore, previous studies reported that elevated RDW levels in pregnancy were associated with the occurrence and severity of preeclampsia^(11,17)^. We were unable to demonstrate any difference in the RDW levels between the three groups.

Leukocytosis is a physiologic change observed in healthy pregnancies secondary to neutrophilia, and WBCs are known to be the mediator of inflammation^(19)^. It has been reported that first trimester leukocytosis is associated with adverse pregnancy outcomes, in particular with preterm delivery^(7)^. Canzoneri et al.,^(20)^ have shown that increased total leukocyte count was associated with both preeclampsia and disease severity. According to their findings, neutrophilia was the only WBC subgroup associated with severe preeclampsia. Moreover, changes in WBC functions occur in addition to the elevated neutrophil levels in women with preeclampsia, and phenotypic and metabolic changes in granulocytes and monocytes have also been shown in *in vivo* studies^(21)^. Our findings are consistent with those of previous studies. Although an increased first trimester WBC count, which is likely due to neutrophilia, is associated with preeclampsia, the counts of eosinophils, basophils, monocytes, and lymphocytes did not differ in preeclamptic women compared with controls. In the present study, we showed that a WBC count >9.55×10^3^/µL was associated with EOPE with 71.4% sensitivity and 70.7% specificity. In addition, a neutrophil count >6.45×10^3^/µL was found to be useful to predict EOPE with 66.7% sensitivity and 74.8% specificity.

Insufficient placental development and remodeling are responsible from obstetric complications, which may be consequences of increased maternal immune system response to the fetal structures. The cause of increased neutrophils and WBCs in maternal blood is most probably due to this altered maternal inflammation. Activated neutrophils and WBCs secondary to the endothelial dysfunction in preeclampsia may be responsible from the increased first trimester levels of WBC and neutrophils.

Platelet indices such as platelet count, MPV, plateletcrit, and PDW are important indicators of platelet activation and thromboembolic events. Thrombocytopenia may be a late laboratory finding of preeclampsia, and low levels are usually associated with more severe disease^(22)^. However, several studies have reported that first trimester platelet count might not be a good marker to predict subsequent preeclampsia^(12,23)^. In our study, the median platelet levels in the first trimester were lower in the control group and higher in the EOPE group, but this finding was not statistically significant. Increased PDW and MPV together with decreased plateletcrit levels have been reported to be associated with preeclampsia^(12)^. However, Monteith et al.,^(2)^ studied the MPV changes in each trimester and the prediction of EOPE by comparing patients with healthy subjects. However, they were unable to determine any significant first trimester MPV value for the prediction of EOPE. In contrast, first trimester MPV levels of >9.95 fL were found to be a valuable marker to predict preeclampsia in a different study^(23)^. According to our findings, we found no correlation between thrombocyte parameters in preeclampsia and disease onset.

NLR and PLT are two other inflammatory markers that can easily be calculated. The prognostic value of increased NLR in cardiovascular diseases has been well described in the literature,^(24)^ and neutrophilia with a stable lymphocyte count leads to an increased NLR in preeclampsia. Several studies have suggested that an elevated NLR might be an effective marker to predict preeclampsia and disease severity^(25,26,27)^. In our study, although the NLR decreased from EOPE to controls, this was not statistically significant, most likely owing to the small number of participants. The consumption of platelets after endothelial injury results in thrombocytopenia in preeclampsia. As a result, researchers have claimed that PLR levels may decrease in preeclampsia. Generally, the results pertaining to PLR changes in preeclampsia are conflicting, with both increased^(26)^ and decreased levels^(11)^ reported in the literature. We were unable to demonstrate any difference between the three groups in terms of either NLR or PLR values. Minor changes in CBC parameters may not have reached statistical significance in the present study to show the changes in the NLR and PLR. Also, increased or decreased levels of neutrophils, lymphocytes, and platelets in each patient may not be enough to show a difference, and several clinical and pathologic events may be responsible for this issue. Although these two parameters are both thought to be correlated with altered inflammation, physicians should be aware of the high false and negative predictive values to predict preeclampsia in clinical practice.

### Study Limitations

Missing and unreliable data due to the retrospective design of the study and the use of a single center are the main limitations of our study. The number of patients was relatively lower in the EOPE group than in the other groups, which may have affected the results. Moreover, CBC test results may have been influenced by several factors such as smoking, drug intake, and concomitant diseases and infections, and these factors may not have been disclosed by some patients.

## Conclusion

In conclusion, we have demonstrated that first trimester leukocytosis and increased neutrophils are the only CBC indices associated with the onset time of preeclampsia. We did not observe any significant difference between the other inflammation markers (MPV, NLR, and PLR) in the EOPE group as compared with the LOPE group and healthy controls. Further well-designed studies with multicenter participation are warranted to ascertain the changes in first trimester CBC parameters in EOPE.

## Figures and Tables

**Table 1:**
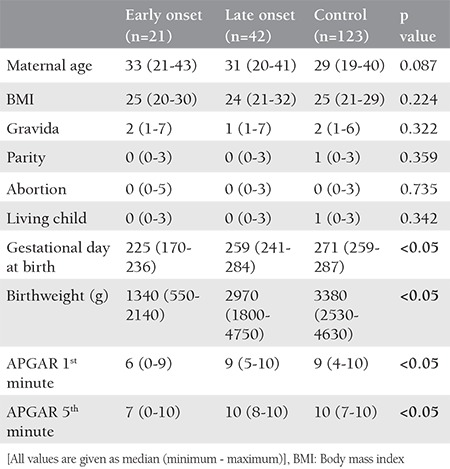
Characteristics of the patients, delivery times, and neonatal findings

**Table 2:**
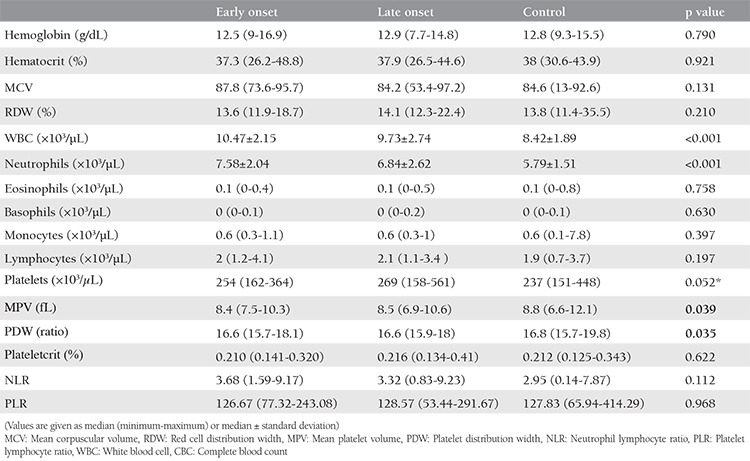
Comparison of the first trimester CBC results

**Table 3:**
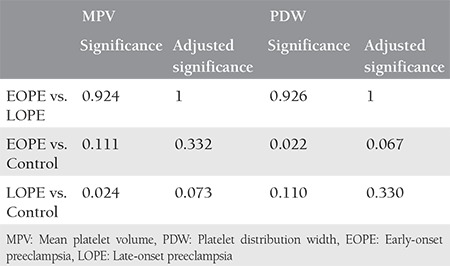
Pairwise comparison results for MPV and PDW (p values)
